# Efficacy of acupuncture for chronic knee pain: protocol for a randomised controlled trial using a Zelen design

**DOI:** 10.1186/1472-6882-12-161

**Published:** 2012-09-19

**Authors:** Rana S Hinman, Paul McCrory, Marie Pirotta, Ian Relf, Kay M Crossley, Prasuna Reddy, Andrew Forbes, Anthony Harris, Ben R Metcalf, Mary Kyriakides, Kitty Novy, Kim L Bennell

**Affiliations:** 1 Centre for Health, Exercise and Sports Medicine, Department of Physiotherapy, School of Health Sciences, Faculty of Medicine Dentistry & Health Sciences, The University of Melbourne, Melbourne, VIC, AUSTRALIA; 2 Department of General Practice, School of Medicine, Faculty of Medicine Dentistry & Health Sciences, The University of Melbourne, Melbourne, VIC, AUSTRALIA; 3 Physiotherapy, School of Health & Rehabilitation Sciences, The University of Queensland, Brisbane, QLD, AUSTRALIA; 4 School of Medicine and Public Health, Faculty of Health, University of Newcastle, Callaghan, Newcastle, NSW, AUSTRALIA; 5 Epidemiology & Preventative Medicine, Faculty of Medicine, Nursing & Health Sciences, Monash University, Melbourne, VIC, AUSTRALIA; 6 Centre for Health Economics, Faculty of Business & Economics, Monash University, Melbourne, VIC, AUSTRALIA

## Abstract

**Background:**

Chronic knee pain is a common and disabling condition in people over 50 years of age, with knee joint osteoarthritis being a major cause. Acupuncture is a popular form of complementary and alternative medicine for treating pain and dysfunction associated with musculoskeletal conditions. This pragmatic Zelen-design randomised controlled trial is investigating the efficacy and cost-effectiveness of needle and laser acupuncture, administered by medical practitioners, in people with chronic knee pain.

**Methods/Design:**

Two hundred and eighty two people aged over 50 years with chronic knee pain have been recruited from metropolitan Melbourne and regional Victoria, Australia. Participants originally consented to participate in a longitudinal natural history study but were then covertly randomised into one of four treatment groups. One group continued as originally consented (ie natural history group) and received no acupuncture treatment. The other three were treatment groups: i) laser acupuncture, ii) sham laser or, iii) needle acupuncture. Acupuncture treatments used a combined Western and Traditional Chinese Medicine style, were delivered by general practitioners and comprised 8–12 visits over 12 weeks. Follow-up is currently ongoing. The primary outcomes are pain measured by an 11-point numeric rating scale (NRS) and self-reported physical function measured by the Western Ontario and McMaster (WOMAC) Universities Osteoarthritis Index subscale at the completion of treatment at 12 weeks. Secondary outcomes include quality of life, global rating of change scores and additional measures of pain (other NRS and WOMAC subscale) and physical function (NRS). Additional parameters include a range of psychosocial measures in order to evaluate potential relationships with acupuncture treatment outcomes. Relative cost-effectiveness will be determined from health service usage and outcome data. Follow-up assessments will also occur at 12 months.

**Discussion:**

The findings from this study will help determine whether laser and/or needle acupuncture is efficacious, and cost-effective, in the management of chronic knee pain in older people.

**Trial registration:**

Australian New Zealand Clinical Trials Registry reference: ACTRN12609001001280

## Background

Chronic knee pain is a common and disabling condition, particularly in people over 50 years of age [1,2]. Knee joint osteoarthritis (OA) is a major cause of knee pain and results in loss of functional independence, psychological impairment and a reduction in the overall quality-of-life of affected individuals. In addition to the personal burden of knee OA, there are substantial direct and indirect health care costs particularly in terms of employment status, productivity and joint replacement surgery, making knee OA a substantial public health problem.

Management of knee OA is primarily focused on treating the pain and disability associated with the condition, with non-pharmacological therapies considered the cornerstone of treatment [3]. Acupuncture is a popular treatment for pain and dysfunction associated with musculoskeletal conditions. A study of 350 rheumatology patients in the United Kingdom revealed that 61% of those with OA had used acupuncture [4]. In clinical practice, a range of health professionals administer or refer patients for acupuncture. In Australia, acupuncture by medical practitioners has been established for 40 years and has gained widespread patient acceptance in the community. We have found that over 80% of surveyed general medical practitioners referred patients for acupuncture [5] and that they generally consider acupuncture to be highly effective and safe [6]. Amongst all complementary and alternative medicine modalities, acupuncture is associated with the highest average annualised expenditure [7]. Thus research to determine its efficacy and cost-effectiveness is warranted.

Acupuncture traditionally involves the insertion of fine needles into specific points of the body. According to the ancient philosophy of traditional Chinese acupuncture, energy circulates in 12’meridians’ located throughout the body. Pain or ill health will result if the meridian energy circulation is blocked. Stimulating the appropriate combination of meridian acupuncture points in the body can restore energy circulation, health, and balance [8]. From a Western medicine perspective, acupuncture is a technique of peripheral sensory stimulation (through activation of peripheral A-delta and C fibres) applied at acupuncture points and/or trigger points that can activate central nervous system pain pathways, release specific pain relieving substances as well as reduce muscle and sympathetic nervous system tonicity [9,10].

In recent years there has been a substantial increase in the number of randomised controlled trials (RCTs) investigating the efficacy of needle acupuncture for chronic knee pain. A recent meta-analysis concluded that while placebo-controlled trials show statistically significant benefits for pain, the benefits are small and unlikely to be clinically relevant [8]. Waiting list-controlled trials however suggest statistically significant and clinically relevant benefits, which may be due to expectation or placebo effects. Accordingly, well-designed rigorous RCTs are needed that control for both placebo and expectation effects in order to evaluate the efficacy of acupuncture for chronic knee pain.

Although acupuncture has been traditionally administered with needles, the use of laser acupuncture has increased because of its pain-free nature and minimal adverse effects [11]. Laser acupuncture involves the application of low-level laser, which is a form of electromagnetic radiation in the visible or infrared region of the light spectrum. These laser devices are manufactured with such low energy densities that photo-chemical changes are elicited without photo-thermal effects. Within these ranges, various energy levels and wavelengths can be used depending on the tissue penetration required. In laser acupuncture, the beam of light generated is applied to the skin at acupuncture points and/or trigger points, similar to the application of needles.

Laser acupuncture effects are thought to alter peripheral afferent nerve stimulation, modulating spinal cord afferent input on second order neurons and enhancing peripheral endogenous opioid analgesia [12] as well as acting via centrally mediated mechanisms. Laser also has effects at the local cellular and tissue level [13] with evidence of modulation of inflammatory processes [14] varying according to laser dosage [15].

In contrast to needle acupuncture, the efficacy of laser acupuncture has been less well studied in the management of chronic knee pain. In general, RCTs have used small sample sizes and variable laser dosages in terms of wavelength, total energy, application sites and number of treatments. In a meta-analysis of eight trials published up until 2006 investigating the short-term efficacy (within 4 weeks) of laser therapy for knee OA, statistically and potentially clinically significant pain-relieving benefits over placebo were found [11]. The effect was greater when only trials evaluating an optimal dosage and administration were included. Since this meta-analysis, two other small studies have been published providing further support for the use of laser acupuncture in chronic knee pain [16,17].

It is not clear whether differences in efficacy exist between laser and needle acupuncture given that there are no direct head-to-head comparisons of needle and laser acupuncture for chronic knee pain., However, in a systematic review, laser but not needle acupuncture offered statistically and clinically significant short-term pain relief over placebo [11]. Similarly, a meta-analysis found that treatment with laser acupuncture had better pain outcomes in the medium-term, although not short-term, than treatment with needle acupuncture in people with non-specific neck pain [18]. These studies suggest that laser acupuncture may be at least as effective, if not more effective, than needle acupuncture.

It is apparent that there is a need for further evaluation of acupuncture in the management of chronic knee pain. This is highlighted by discrepancies in recommendations by clinical guidelines – the Osteoarthritis Research Society International currently recommend acupuncture [3], the United Kingdom NICE clinical guidelines could not give a firm recommendation due to insufficient evidence [19] and the American College of Rheumatology [20] conditionally recommend acupuncture but only for patients with moderate to severe pain who are candidates for joint replacement but are unwilling/unable to have surgery. Furthermore, there is limited information about the maintenance of acupuncture effects with no studies reporting a follow-up longer than three months.

In designing appropriate clinical trials, a methodological concern in acupuncture trials has been the difficulty in having a true placebo control group given pre-existing participant expectations, particularly where the treatment itself is multidimensional involving therapist-patient interaction as well as an intervention [21]. A meta-analysis has confirmed a significant placebo effect for the treatment of pain in knee OA that varies in size depending on the intervention [22]. For acupuncture, the placebo effect size (mean 0.71 (95% confidence interval 0.53, 0.90)) was higher than the overall effect size for all placebo treatments (0.51 (0.46, 0.55)). As with many treatments, higher patient expectation has been shown to be associated with greater improvements following acupuncture treatment. A pooled analysis of four clinical trials of acupuncture found that after completion of treatment, the odds ratio for response between patients considering acupuncture an effective or highly effective therapy and patients who were more sceptical was 1.67 (95% confidence interval 1.20-2.32) [23]. The use of “sham needle” acupuncture as a placebo control, whereby needling is performed superficially or at non-acupuncture sites, is controversial given that such a procedure may still produce biological responses and hence may not be truly placebo. Blinding of clinicians administering needle acupuncture is also problematic. Such methodological difficulties can be overcome in trials of laser acupuncture as it is possible to successfully blind both participants and clinicians by using specially designed laser machines [24].

First described by Zelen in the New England Journal of Medicine in 1979 [25], Zelen design trials are a means of reducing bias by minimizing participant expectations in a treatment trial where the knowledge of the intervention itself may influence the study outcome (Hawthorne effect) in the control group. This is done by enrolling participants into an observational study but covertly randomising participants following their recruitment into a treatment trial (post-randomised consent). The control group remains unaware of the treatment being evaluated [26]. Such a design has been used successfully in a study comparing a physiotherapy treatment of exercise and knee taping to standard non-physiotherapy treatment in people with predominant patellofemoral OA [27]. A potential disadvantage of the Zelen design can be a higher crossover rate than a usual RCT which may dilute treatment effects in an intention-to-treat analysis. However, a review of 58 RCTs using the Zelen design found that while most trials (n = 41) experienced some crossover from one group to the other (median crossover = 8.9%, interquartile range 2.6% to 15%) the rate was usually within acceptable limits [26].

Thus, we are currently conducting a Zelen design RCT to investigate the efficacy of needle and laser acupuncture administered by experienced medical acupuncturists in people with chronic knee pain and to evaluate maintenance of effects over the longer term. We will also evaluate the cost-effectiveness of needle and laser acupuncture, as well as explore whether psychosocial measures are associated with changes in pain, physical function and health-related quality of life following acupuncture treatment. This paper describes the protocol for this ongoing trial.

The primary hypotheses are that:

H1: Laser acupuncture will result in significantly greater improvements in pain (as measured overall via numerical rating scale (NRS)) and physical function (as measured via Western Ontario and McMaster (WOMAC) Universities Osteoarthritis Index) than sham laser acupuncture at 12 weeks.

H2: Laser and needle acupuncture will result in significantly greater improvements in pain (as measured overall via NRS and physical function (as measured via WOMAC) than no treatment at 12 weeks.

H3: Laser acupuncture will result in significantly greater improvements in pain (as measured overall via NRS) and physical function (as measured via WOMAC) than needle acupuncture at 12 weeks.

The secondary hypotheses are that:

H4: Sham laser acupuncture will result in significantly greater improvements in pain (as measured overall via NRS) and physical function (as measured via WOMAC) than no treatment at 12 weeks.

H5: Laser, sham laser and needle acupuncture will result in significantly greater improvements in pain (as measured overall via NRS) and physical function (as measured via WOMAC) than no treatment at 12 months, while laser acupuncture will result in significantly greater improvements than needle acupuncture.

H6: Laser, sham laser and needle acupuncture will result in significantly greater improvements in pain (as measured on standing and walking via NRS and by WOMAC), physical function (as measured via NRS) and health-related quality of life than no treatment at 12 weeks and 12 months.

H7: Laser acupuncture will result in significantly greater improvements in pain (as measured on standing and walking via NRS and by WOMAC), physical function (as measured via NRS) and health-related quality of life than needle acupuncture at 12 weeks and 12 months.

H8: A greater proportion of people receiving laser, sham laser and needle acupuncture will report global overall improvements compared to no treatment at 12 weeks and 12 months.

H9: A greater proportion of people receiving laser acupuncture will report global overall improvements compared to needle acupuncture at 12 weeks and 12 months.

## Methods/Design

### Trial design

**Figure 1:**
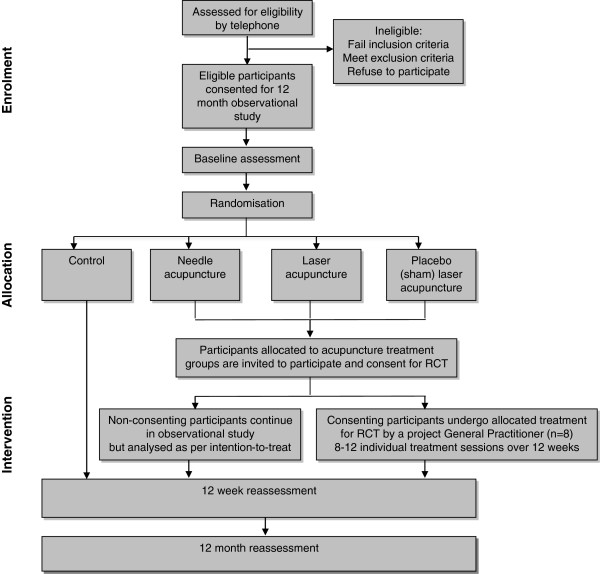
Flow diagram of study protocol.

A two-stage Zelen design RCT [25,28] which conforms to CONSORT [29] and STRICTA guidelines [30] for acupuncture studies (Figure 1). 

### Participants

A total of 282 men and women with chronic knee pain have been recruited from the community in metropolitan Melbourne and regional Victoria, Australia. A number of recruitment strategies were used including (i) advertisements in local clubs, community centres, newspapers, Arthritis Australia and University websites, University staff newsletters, radio, television, and a social networking site; (ii) placement of brochures and study posters in medical and physiotherapy clinics; and (iii) presentations about knee OA in the local community by the researchers.

People were eligible to participate if they (i) were aged >50 years; (ii) had a history of knee pain of >3 months duration; (iii) reported knee pain on most days of the past month; (iv) reported an average knee pain severity over the past month of ≥ 4 out of 10 on an 11-point NRS and; (v) reported morning knee stiffness of <30 minutes duration.

Exclusion criteria included: (i) history of any systemic arthritic condition; (ii) history of knee arthroplasty on the most painful knee; (iii) wait-listed for any knee surgery for either knee; (iv) history of any knee surgery in previous 6 months; (v) any other condition affecting lower limb function (eg trauma, malignancy, neurological condition); (vi) history of any knee injection in past 6 months (eg cortisone, hyaluronic acid); (vii) current use of oral or injectable anticoagulant medication; (viii) use of acupuncture in past 12 months; (ix) any bleeding disorder; (x) allergy to light; (xi) referral to pain clinic or use of morphine or pethadine within past 6 months; (xii) any other medical condition precluding participation in the trial (eg kidney or liver disease, deep vein thrombosis); (xiii) knee pain subject to compensation claim or; (xiv) unable to give written informed consent.

Although both knees could undergo treatment as part of the project, only the most symptomatic knee as nominated by the participant at enrolment was evaluated.

### Procedure

Figure 1 depicts the trial protocol. Eligibility of prospective participants was determined over the telephone by an investigator (MK) not involved in assessment or treatment of participants. Ethical approval was obtained from the University of Melbourne Human Research Ethics Committee (HREC No. 0931840). All participants provided written informed consent. Initially, all participants consented to participate in a longitudinal observational natural history study of chronic knee pain, involving assessments at baseline, 12 weeks and 12 months. All assessments, comprised solely of questionnaires, are completed by participants at home and mailed back to the University of Melbourne, where data is entered by a blinded assessor (MK).

Upon receipt of completed baseline questionnaires, an investigator (KN) accessed the computerised covert randomisation schedule to reveal whether the participant was allocated either (i) laser acupuncture (could be either real laser or sham laser but the investigator and participant are blinded to this information); (ii) needle acupuncture or; (iii) no treatment (control group). Participants randomised into the no treatment arm continue as consented in the natural history observational cohort for the 12 month duration of the study and did not undergo (or are made aware of) any intervention as part of the trial. Participants randomised to receive either laser or needle acupuncture were telephoned by an investigator (KN) and offered the opportunity to undergo the allocated intervention and contribute their data to the trial. Participants allocated to the laser groups were informed they may receive either real laser or sham laser and that this has been randomly pre-determined and would not be revealed to them until the completion of the 12 month assessments. Participants willing to participate in the intervention provided informed consent to do so at this point. Participants who choose not to participate in the intervention continued as initially consented in the natural history observational cohort for 12 months, however their data will be analysed as per intention-to-treat.

Participants who were randomised to receive acupuncture had treatment visits arranged with a project general practitioner (GP) for treatment over 12 weeks. All study participants, including those allocated to the no treatment group and those who were randomised to receive treatment but declined to do so, undergo re-assessment at 12 weeks and again at 12 months.

### Randomisation and allocation concealment

The randomisation schedule was prepared by the study biostatistician (AF). All eligible participants were consecutively randomised into either the (i) real laser acupuncture; (ii) sham laser acupuncture; (iii) needle acupuncture or; (iv) no treatment group. Allocation was randomised within random permuted blocks of six to twelve generated a priori and stratified according to treating GP so that all GPs delivered approximately equal numbers in each acupuncture group to control for therapist variation. An electronic password-protected spreadsheet that contained concealed acupuncture group allocation was prepared by a researcher (BM) with no other involvement in the study, and held by an investigator (MP) not involved in recruitment, assessment or treatment. The patient code numbers for the randomised laser treatment arms were entered into the study laser machines by an independent biomedical engineer, in order to permit blinding of both the treating GPs and the participants.

### Blinding

Laser and sham laser participants were blinded to treatment allocation, as were the GPs administering laser and sham laser acupuncture, as the same machine provided both interventions and the participant code (entered by the GP) determined whether the machine actually outputted laser or not [24]. Participants in the no treatment group are unaware that the treatment groups exist and that their data will be used for comparison (despite having consented to their longitudinal data being used for other unspecified research questions as determined by the researchers). Only the participants undergoing needle acupuncture and the GP provider were unblinded to their treatment group allocation.

### Interventions

This trial employed a combined Western and Traditional Chinese Medicine style of acupuncture, utilizing standardised acupuncture points. Such an approach reflects Australian medical acupuncture training and clinical practice. Participants were treated by experienced GPs who are also members of the Australian Medical Acupuncture College. These GPs have completed a standardised University-delivered acupuncture training course, undertaken formal accreditation by examination and a minimum period of supervised clinical experience, and are registered as medical practitioner acupuncturists by the Medical Board of Victoria. Medical acupuncturists were selected for this study to minimise potential confounder effects related to using health practitioners with varying qualifications, clinical experience and expertise. A total of 8 GPs in 9 locations around metropolitan Melbourne and regional Victoria were involved (Table 1). The GPs had an average of 33.3 years of clinical practice and 19.6 years of medical acupuncture experience. None had less than 10 years acupuncture experience.

**Table 1:** Descriptive characteristics of the general practitioners who provided acupuncture treatment

**General practitioner**	**Gender**	**Location(s)**	**Years of medical practice**	**Years of acupuncture experience**
1	Male	Bacchus Marsh	23	14
		East Melbourne		
2	Male	Anglesea	30	24
3	Male	Wendouree	48	30
4	Female	Glen Waverley	25	18
5	Male	Mildura	20	10
6	Male	Traralgon	25	12
7	Male	Malvern	50	19
8	Male	Bendigo	45	30

The GPs attended a four-hour training session run by an investigator (IR) who is an experienced medical acupuncturist, to standardise all aspects of the treatment protocol. This same investigator maintained regular contact with the GPs throughout the trial to answer any treatment-related questions and ensure compliance. Each GP was provided with a detailed treatment manual describing administration of the acupuncture interventions as well as the trial protocol. GPs were supplied with a pre-coded laser machine (custom-designed to deliver both real and sham laser) and standardized needles required for administering the acupuncture interventions.

Acupuncture treatments (approximately 20 minutes in duration) were administered once or twice weekly (at the GP’s discretion) in the GP’s rooms for 12 weeks, with a minimum of 8 and a maximum of 12 treatments delivered. Participants did not pay for treatment and the treating GPs were reimbursed for their time on a per patient basis.

The treatment protocol permitted the GPs to treat participants as they would treat patients in their normal practice. They were allowed to select from a standardized set of acupuncture points around the knee, as well as distal points on the intervention side (Table 2). Other points could be used at the GP’s discretion depending on the cause(s) and site of the pain evident at clinical examination. These included points indicated by Traditional Chinese Medicine diagnoses in relation to Zang-Fu organ dysfunction, associated meridian and deep channel connections and ear points. Because of varying participant sensitivity, the initial treatment session permitted a maximum of six points in total, including four points on the study limb and two other points chosen as per protocol. In subsequent treatment sessions, further points were added as clinically indicated by participant response to treatment, as well as local tenderness, muscle tightness and/or swelling. Over subsequent treatments, acupuncture points were varied according to changes in participant symptoms and point reactivity. The 12-week treatment period allowed for patient attendance flexibility (a break of up to four weeks between visits was deemed acceptable for participants going away on holidays etc.), as well as for strong acupuncture responders to have several days settling time as necessary. Visits were scheduled twice per week if necessary, but weekly visits were generally recommended. Treatment was considered complete when pain and function had returned to normal, however a minimum of 8 treatments was required. Laser acupuncture interventions were administered with the patient positioned either supine or sitting over the edge of the treatment couch. Treating GPs completed standardized treatment notes after each acupuncture session. Information recorded included the acupuncture points treated and any adverse events reported by the patient from the previous session.

**Table 2:** Framework of acupuncture points that could be selected by general practitioners when administering acupuncture treatments (laser and needles)

**Location**	**Acupuncture points**
Local points	SP9, 10
	ST34, 35, 36
	LR7, 8, 9
	KI10
	BL39, 40, 57
	GB34, 35, 36
	Local extra points in the hamstring muscles
Distal points	ST40
	LR3
	SP6
	GB41
	BL60
Segmental points	BL21, 22, 23
	GB30, 31
Non-segmental and general points	Ear Knee point
	DU20
	Li11
	GV14
	BL11

#### Needle acupuncture

Patients randomised to the needle acupuncture treatment underwent treatment lying down with needles inserted at the selected acupuncture points. Single-use Sierin needles (0.25x40mm) were used. The needles were inserted and left in place with the participant to rest. Needles were disposed of into a bedside sharps container at the completion of treatment.

#### Laser and placebo laser acupuncture

Laser acupuncture was administered at the selected acupuncture points using a Acupak P/L (Melbourne) laser machine that was specially manufactured for use in this trial [24]. The laser machines were standard Class 3B laser devices that are licensed by the Australian Therapeutic Goods Association with an output of 10 mW. Energy output was 0.2 J per acupuncture point. Laser strength was checked by the manufacturer (a biomedical engineer) at study commencement, halfway through the study and will be repeated at study completion. Laser strength testing is a routine procedure in laser manufacture and maintenance and is done with a standard laser testing device. The trial laser machine had a small red non-laser light source arising from inside the probe tip that lit up when the probe was in both treatment mode and sham mode (no output). This acted as a decoy to the treatment beam, which is invisible. Therefore, neither the treating GP nor the participant could detect any difference between the real or sham laser treatment being applied. The trial laser machines were programmed by the manufacturer. At each treatment session, the GP typed the participant’s study code into the keypad of the machine, and the machine automatically delivered either laser acupuncture (ie laser output) or sham laser acupuncture (ie no laser output) according to the programmed randomisation schedule.

### Outcome measures

Table 3 summarises the range of measures that are being collected as part of this study. The majority of outcomes are measured at baseline, 12 weeks and 12 months in all participants (irrespective of group allocation). All outcomes are collected via self-report questionnaire and mailed back to the investigators.

**Table 3:** Summary of measures collected as part of the study

**Primary outcome measures**	**Data collection instrument**
Average overall pain in past week	11-point numerical rating scale (NRS)
Physical function in past 48 hours	Western Ontario McMaster Universities (WOMAC) Osteoarthritis Index 3.1 Likert version
**Secondary outcome measures**	
Average pain on i) walking and ii) standing in past week	11-point NRS
Pain over past 48 hours	WOMAC Osteoarthritis Index 3.1 Likert version
Average restriction to daily activity in past week	11-point NRS
Participant global rating of change in i) pain ii) physical function and iii) overall.	5-point ordinal scales (12 weeks and 12 months)
Health-related quality of life	Assessment of Quality of Life Instrument (AQoL II)
	12-item Short Form Health Survey (SF-12)
**Other measures**	
Psychosocial parameters	Arthritis Self-Efficacy Questionnaire
	Illness Perception Questionnaire Revised (IPQ-R)
	Medical Outcomes Study (MOS) Social Support Survey
	9-item Patient Health Questionnaire (PHQ-9)
	Revised Health Hardiness Inventory
Physical activity	Physical Activity Scale for the Elderly (PASE)
Use of health care/home assistance and work absences	Log book (retrospectively for prior 4 weeks)
Adverse events	Open-ended questioning (12 weeks)
Treatment session attendance	General practitioner treatment records (12 weeks)

All measures are recorded at baseline, 12 weeks and 12 months unless otherwise stated.

Age, gender, height, weight, duration and nature (unilateral/bilateral) of knee symptoms and previous treatment for knee joint problems were obtained by questionnaire at baseline for descriptive purposes.

#### Self-reported pain and physical function

The primary pain outcome is average overall knee pain over the past week. This is assessed using an 11-point NRS with terminal descriptors of “no pain” and “worst pain possible”. Similar scales are also being used to assess average pain on standing and on walking over the past week as secondary outcomes. Pain is also assessed, along with physical function, using the disease-specific WOMAC Osteoarthritis Index [31]. The physical function subscale, which comprises 17 questions, is the primary outcome measure for self-reported physical function. A secondary measure of physical function is a NRS assessing average restriction to daily activities over the past week with terminal descriptors of “no restriction” and “maximum restriction possible”.

At the 12 week and 12 month assessments, participants rate their perceived change in a) pain; b) physical function and; c) overall, compared to baseline on a five-point ordinal scale (1-much worse to 5-much better). Scales of this kind are frequently used as an external criterion for comparison with changes in scores of other outcomes [32]. Measuring participant-perceived change using a rating of change scale has been shown to be a clinically relevant and stable concept for interpreting truly meaningful improvements from the individual perspective [33].

#### Health-related quality of life

Health-related quality of life is measured using the Assessment of Quality of Life instrument version two (AQoL II). The AQoL II has 20 questions that cover six dimensions of health-related quality of life including independent living, social relationships, physical senses, coping, pain and psychological wellbeing. The AQoL has strong psychometric properties and is more responsive than other more widely used scales [34,35]. It produces a single utility index that ranges from −0.04 (worst possible health-related quality of life) to 1.00 (full health-related quality of life).

The 12-item Short Form Health Survey (SF-12) is also being used to measure health-related quality of life [36]. The SF-12 is a self-administered questionnaire that was designed to measure patient outcomes in medical practice and clinical research and to evaluate the effectiveness of health interventions. The 12 questions in the SF-12 measure an individual’s perceived health across eight domains: physical functioning; role limitations because of physical health problems; bodily pain; general health perceptions; energy/fatigue; social functioning; role limitations because of emotional problems; and general mental health. The SF-12 is scored so that a high score indicates better functioning. Scoring algorithms are applied to individual item responses to produce the composite Physical Component Summary (PCS) and Mental Component Summary (MCS) scores. The PCS and MCS scores have a range of 0 to 100 and were designed to have a mean score of 50 and a standard deviation of 10; scores greater than 50 represent above average health status.

#### Psychosocial measures

A range of psychosocial measures are being collected in order to evaluate whether psychosocial parameters are associated with changes in pain, physical function and health-related quality of life observed with acupuncture.

i) Arthritis Self-Efficacy Scale is used to measure self-efficacy. It has three subscales that assess self-efficacy for control of pain management (5 questions), physical function (9 questions) and other arthritis symptoms (6 questions). Each question is rated on a 10-point NRS from 1 (“very uncertain”) to 10 (“very certain”). Prior studies have supported both the reliability and validity of this scale [37].

ii) Illness Perception Questionnaire Revised (IPQ-R) is used to measure participant’s perceptions of their arthritis [38]. It comprises 38 items, each rated on a 5-point Likert scale, ranging from “strongly disagree” to “strongly agree”. The IPQ-R assesses numerous dimensions including identity, timeline acute/chronic, timeline cyclical, consequences, personal control, treatment control, illness coherence, emotional representations. It has demonstrated reliability and validity [38].

iii) Medical Outcomes Study (MOS) Social Support Survey is used to measure the perceived social support available to an individual. It comprises six questions that ask respondents to indicate how often each of six different types of support is available to them. Responses are scored on a five-point scale ranging from “all of the time” to “none of the time”. This measure of social support has been shown to have adequate validity and reliability [39].

iv) Patient Health Questionnaire (PHQ) is a self-administered version of the PRIME-MD diagnostic instrument for common mental disorders [40] . This trial is utilizing the PHQ-9 [41], which is the depression module that scores each of the 9 DSM-IV criteria [42] as “0” (not at all) to “3” (nearly every day).

v) Revised Health Hardiness Inventory comprises 24 items and is used to measure health hardiness (control, commitment, and challenge) in individuals with actual health problems. Respondents indicate on a 5-point scale the degree to which they agree with the statement, ranging from “strongly disagree” to “strongly agree”. This questionnaire has adequate psychometric properties, and is a reliable and useful instrument to assess perspectives towards one’s own health [43].

#### Physical activity levels

Habitual physical activity is measured using the Physical Activity Scale for the Elderly (PASE), a self-report questionnaire that has been shown to be reliable, valid and sensitive to change in people with knee OA [44,45]. It records both the level and type of recreational and occupational physical activity undertaken by participants over the previous week. The PASE was developed and validated in samples of older adults (age 55+ years) [46].

#### Use of health care, home assistance and work absences

Information on health care use (e.g. visits to hospitals, medical practitioners, other health professionals, use of investigative procedures, prescription and non-prescription drugs) and the need for paid and/or unpaid home assistance over the previous four weeks will be collected via a log-book at baseline, 12 weeks and 12 months. Participants are also asked to record any absences from paid employment (if applicable) and/or unpaid work (eg household duties, caring for family/friends) because of their health. The log-book utilises a checklist format to minimize respondent burden and to ensure high quality data is obtained with minimal data loss.

#### Adverse events

Adverse events were monitored via open-ended questioning by the acupuncturist at each treatment session and recorded in the treatment notes. In addition, participants receiving acupuncture treatment were asked at 12 weeks whether they experienced any adverse effects of treatment via open-ended questioning.

#### Treatment session attendance

Participant adherence was measured by recording the number of treatment sessions attended (out of a maximum number of 12) in the GP treatment notes.

### Sample size calculations

Our primary endpoints are average overall knee pain measured on a NRS and physical function measured via WOMAC at 12 weeks. The minimum clinically important difference to be detected in OA trials is a change in pain of 1.8 units (on NRS) [47] and a change of 6 physical function units on the WOMAC (out of 68) [48]. Calculations are based on an analysis of covariance (ANCOVA) adjusting for baseline of the outcome variable, and assume a between-patient standard deviation of 3.0 units for pain and 12.0 units for WOMAC [49]. Sample size calculations for our particular study design need to take into account four aspects: clustering effects resulting from treatment of patients by the same therapist for comparisons with the control arm (hypothesis H2); dilution of the treatment effect resulting from the Zelen design [50]; precision gain from baseline adjustment; and loss to followup. Initially assuming no clustering effects and a regular two-arm parallel RCT design with complete followup, 33 patients per arm would be required to detect the above differences in pain and function with 80% power at a two-sided 5% significance level, using ANCOVA with assumed baseline to 12 week correlations of 0.50 for pain and 0.70 for function [49]. Adopting a conservative intra-therapist correlation of 0.10 inflates the sample size to 40 patients per arm across a total of 10 therapists. Assuming a conservative 15% non-consent rate for patients randomised to any of the three treatment arms increases the sample size by 1/(1–0.15)^2^ = 38%, and finally, allowing for 15% loss to followup, inflates the total sample size to 66 patients per arm, which we round up to 70 per arm, or 280 patients in total. Assessment of hypotheses H1 and H3 involve within-therapist comparisons only and therefore the precision loss from clustering by therapist does not apply, yielding greater power (90%) for these comparisons.

### Statistical analyses

The primary analysis of the data will be undertaken using the principle of intention-to-treat with all randomised patients included in the analyses. Demographic and clinical characteristics, as well as baseline data, will be presented to assess the baseline comparability of the intervention groups. Descriptive statistics will be presented for each group as the mean change (standard deviation, 95% confidence intervals) in the outcomes from baseline to each time point. Differences in mean change from baseline to each time point will be compared between groups using random effects linear regression modelling adjusting for baseline levels of the outcome measure and using the treating therapists as random effects to account for clustering of outcomes by therapists. Model assumptions will be checked by standard diagnostic plots. Improvement between acupuncture groups (based on the perceived ratings of change) will be compared using logistic regression with random effects as above and presented as odds ratios with 95% confidence intervals. Multiple imputation methodology will be employed to account for missing data [51]. No statistical adjustment will be made for multiple testing. All tests will be two sided and carried out at the 5% level of significance.

As part of the secondary analyses of the primary outcomes at 12 weeks and 12 months, we plan to conduct a separate analysis to estimate the effect of treatment in the hypothetical scenario of full adherence to the randomised treatment intervention. Analytical methods for this will utilise instrumental variables methodology involving two stage least squares estimation [52].

### Economic evaluation

The economic evaluation will be conducted from the perspective of the Australian health care system and the individual patient. Standard methods of economic evaluation alongside a clinical trial will be used to evaluate the differences in resource use and health outcomes at the end of treatment and at 12 months. The primary economic evaluation will take the form of a cost effectiveness study with a range of outcome measures including the incremental cost per extra person with a clinically significant improvement in pain and function and per extra quality adjusted life years (QALYs) for laser acupuncture compared to either sham or needle acupuncture. The difference in QALYs will be calculated in the primary analysis as the difference in the area under the curve for AQoL scores over the duration of 12 months. Differences in mean change from baseline for the AQoL to each time point will be weighted by the time from baseline using generalised linear regression modelling adjusting for baseline levels of the AQoL to construct QALYs, and then compared between groups. Differences in the mean cost between groups will be calculated using generalised linear regression modelling. Incremental cost per QALY will be calculated as the ratio of the difference in mean cost to the difference in mean QALYs with 95% confidence intervals calculated using Fieller's theorem.

### Timelines

The application for project funding was successful in December 2008 and funding commenced in August 2009. Ethics approval was obtained from the Human Research Ethics Committee of the University of Melbourne in July 2009. Recruitment commenced in February 2010 and was completed in December 2011. The trial is due for completion in December 2012 when all participants will have completed 12-month follow-up.

## Discussion

This paper has presented the theoretical rationale, as well as the protocol, for an ongoing Zelen design RCT that is testing the efficacy of a combined Western and Traditional Chinese Medicine style of acupuncture for managing chronic knee pain and disability symptoms in older people. The findings of this study will help determine whether laser and/or needle acupuncture are efficacious in relieving knee pain and/or improving physical function. This study will also determine whether effects of acupuncture can be maintained over the longer term, and whether psychosocial factors influence treatment outcomes. Importantly, the use of the Zelen design will minimize the bias typically encountered in traditionally designed RCTs where participant expectations may influence study outcomes.

## Competing interests

The authors declare that they have no competing interests.

## Authors’ contributions

RSH, PM, KMC and KLB conceived the project and PM is leading the co-ordination of the trial. RSH, PM, KMC, MP, IR, PR, AF, AH and KLB developed the protocol and procured the project funding. IR and MP designed the acupuncture program and IR trained the GPs. AF performed the sample size calculations and designed the statistical analyses. AH is leading the collection and analysis of the cost-effectiveness data. MK recruits and screens the participants and performs data entry, while KN randomises participants to groups and is the liaison for the treating GP’s and participants. RSH and KLB wrote the first draft of the manuscript. RSH wrote the final draft of this manuscript. All authors participated in the trial design, provided feedback on drafts of this paper and read and approved the final manuscript.

## Pre-publication history

The pre-publication history for this paper can be accessed here:

http://www.biomedcentral.com/1472-6882/12/161/prepub
